# Oncolytic Viruses and Cancer, Do You Know the Main Mechanism?

**DOI:** 10.3389/fonc.2021.761015

**Published:** 2021-12-22

**Authors:** Wesam Kooti, Hadi Esmaeili Gouvarchin Ghaleh, Mahdieh Farzanehpour, Ruhollah Dorostkar, Bahman Jalali Kondori, Masoumeh Bolandian

**Affiliations:** ^1^ Applied Virology Research Center, Baqiyatallah University of Medical Sciences, Tehran, Iran; ^2^ Department of Anatomical Sciences, Faculty of Medicine, Baqiyatallah University of Medical Sciences, Tehran, Iran; ^3^ Baqiyatallah Research Center for Gastroenterology and Liver Diseases (BRCGL), Baqiyatallah University of Medical Sciences, Tehran, Iran

**Keywords:** oncolytic virus, cancer immunotherapy, cancer vaccine, targeted treatment, immune checkpoint

## Abstract

The global rate of cancer has increased in recent years, and cancer is still a threat to human health. Recent developments in cancer treatment have yielded the understanding that viruses have a high potential in cancer treatment. Using oncolytic viruses (OVs) is a promising approach in the treatment of malignant tumors. OVs can achieve their targeted treatment effects through selective cell death and induction of specific antitumor immunity. Targeting tumors and the mechanism for killing cancer cells are among the critical roles of OVs. Therefore, evaluating OVs and understanding their precise mechanisms of action can be beneficial in cancer therapy. This review study aimed to evaluate OVs and the mechanisms of their effects on cancer cells.

## Background

Millions of individuals are affected by cancer annually. Cancer is considered the leading cause of death and the most important barrier to the increase in life expectancy in the twenty-first century. In 2018, 18.1 million new cancer cases (17.0 million cancer cases excluding non-melanoma skin cancers) were reported. The mortality due to cancer in 2018 was 9.6 million (9.5 million, excluding non-melanoma skin cancers) (1). Significant developments in cancer treatment started in 1900. The achievements of this progress include the development of diagnostic, surgery, chemotherapy, hormone therapy, gene therapy, and cell therapy methods. Regardless of these advancements, human is still incapable of combating cancer, as none of the identified treatment methods could be used in all stages of cancer (2). Many of cancer patients experience a relapse of disease progression regardless of the primary response to treatment.

Furthermore, complete resection of the tumor is difficult or impossible in many cases (3). Immunotherapy has evolved as a practical treatment choice against malignant diseases during the past decades. Studies in oncolytic virotherapy (OVT) developed in the early twentieth century as an observational science for the cases of spontaneous regression of tumors were reported due to infection with specific viruses (4).

Oncolytic viruses (OVs) include a group of viruses that selectively affect and kill malignant cells, leaving the surrounding healthy cells unaffected. OVs have direct cytotoxic effects on cancer cells and augment host immune reactions and result in the destruction of the remaining tumoral tissue and establish a sustained immunity (5). Indeed, OVs function in four ways against tumor cells, including oncolysis, antitumor immunity, transgene expression, and vascular collapse (6). Regarding the fact that cancer cells are developed to avoid detection and destruction by the host immune system and also to resist apoptosis, which are the critical responses of normal cells in limiting viral infections, OVs can kill cancer cells through a spectrum of actions ranging from direct cytotoxicity to induction of immune-mediated cytotoxicity. OVs can also indirectly destroy cancer cells by destroying tumor vasculature and mediating antitumor responses (7). Furthermore, in order to augment the therapeutic characteristics, modifications in OVs by genetic engineering such as insertions and deletions in the genome have been employed in many investigations; thus, additional antitumor molecules can be delivered to cancer cells and effectively bypass the widespread resistance of single-target anticancer drugs (8)

It should be noted that the use of OVs in cancer therapy was limited due to the pathogenicity and toxicity of these viruses in human cases. Recent advancements in genetic engineering have optimized the function of OVs through genetic modifications and therefore have become the issue of interest in OVT (9). Each virus tends to a specific tissue, and this tendency determines which host cells are affected by the virus and what type of disease will be generated. For instance, rabies, hepatitis B, human immunodeficiency virus (HIV), and influenza viruses affect neurons, hepatocytes, T lymphocytes, and respiratory tract epithelium, respectively. Several naturally occurring viruses have a preferential but not exclusive tendency towards cancer cells. This issue is more attributed to tumor cell biology compared to the biology of the virus.

OVs are generally categorized into two groups. One group is preferentially replicated in cancer cells and is not pathogenic for normal cells due to the increased sensitivity to the innate immune system’s antiviral signaling or dependence on the oncogenic signaling pathways. Autonomous parvovirus, myxoma virus (MYXV; poxvirus), Newcastle disease virus (NDV; paramyxovirus), reovirus, and Seneca valley virus (SVV; picornavirus) are categorized in this group. The second group of OVs includes viruses that are either genetically modified for purposes including vaccine vectors such as mumps virus (MV; paramyxovirus), poliovirus (PV; picornavirus), and vaccinia virus (VV; poxvirus), or genetically engineered through mutation/deletion of genes required for replication in normal cells, including adenovirus (Ad), Herpes simplex virus (HSV), VV, and vesicular stomatitis virus (VSV; rhabdovirus) (10).

Furthermore, the mutation in cancer cells, drug adaptation, resistance, and cell immortality were effective in the initiation and speed of viral dissemination. Today, researchers are trying to discover and identify a new generation of OVs to save more patients’ lives from cancer. Evaluation of OVs and identification of the exact mechanism of action of these viruses can be helpful in this way (11). This review study aimed to evaluate OVs and their mechanism of action against cancer cells.

## Methodology

The key terms in the literature search included oncolytic virus, cancer, immunotherapy, innate immunity, adaptive immunity, virotherapy, viral therapy, oncolytic, and virus were searched in international databases, namely, Web of Science, PubMed, and Scopus from 2004 to 2021. The inclusion criterion was the evaluation of viruses using standard *in vivo* and *in vitro* laboratory methods. Exclusion criteria were lack of access to full text articles and incomplete description or assessment of diseases other than cancers.

## Results

The primary search yielded 1,450 articles. Finally, 47 articles were included in the review after eliminating irrelevant and duplicate studies. The characteristics of the 47 included articles are presented in **Table 1**, performed from 2004 to 2021. The OV families assessed in the studies included Ad, MV, PV, NDV, SFV, HSV, VV Reovirus, and bovine herpesvirus (BHV). The most commonly assessed virus was adenovirus (Ad) (n = 15), followed by the herpesvirus (HSV) (n = 12) and measles virus (MV) (n = 7). The least assessed viruses were BHV, SFV, and Reovirus (n = 1).

**Table 1:** The collective studies on OVs.

Virus	Cancer	Model	Effects	Mechanism	References
Adenovirus	Head and neck squamous cell carcinoma	Murine	Ad-derived IL-12p70 prevents the destruction of HER2.CAR-expressing T cells at the tumor site.	Enhanced antitumor effects of HER2 CAR T cells by CAd12_PDL1 Controlling of primary tumor growth and metastasis.	Shaw et al., 2017 (12)
	Renal cell carcinoma	Murine	HRE-Ki67-Decorin suppressed tumor growth and induced decorin expression in the extracellular matrix (ECM) assembly.	An effective anticancer treatment strategy may be chimeric HRE-Ki67 promoter-regulated Ad carrying decorin.	Zhang et al., 2020 (13)
	Lung cancer stem cell (LCSC)	Murine	Tumor necrosis factor (ZD55-TRAIL) increased cytotoxicity and induced A549 sphere cells apoptosis through a mitochondrial pathway	Treatment of lung cancer is possible by targeting LCSCs with armed oncolytic adenovirus genes.	Yang et al., 2015 (14)
	Leukemia	Murine	Induction of autophagic cell death Enhanced cell killing in primary leukemic blasts	Significant autophagic cell death	Tong et al., 2013 (15)
	Breast cancer	Murine	Tumor killing due to Sox2 and oct4 expression and Hoechst 33342 exclusion CD44+CD24−/low cells	A positive effect against advanced orthotopic was that CD44+CD24−/low-derived tumors were observed.	Eriksson et al., 2007 (16)
	Breast cancer	Murine	Delta24 can replicate and help the E1-deleted adenovector replicate in cancer cells	Spontaneous liver metastasis with Delta 24 virus therapy alone was less reduced than in combination with TRAIL gene therapy.	Guo et al., 2006 (17)
	Liver cancer stem-like cells	Murine	Significant apoptosis Inhibition angiogenesis in xenograft tumor tissues Inhibition of the propagation of cells occurred due to GD55	GD55 had a higher effect in suppressing tumor growth than oncolytic adenovirus ZD55.	Zhang et al., 2016 (18)
	B16F10	Murine	Infiltration of effector CD4+ and CD8+ T cells Increasing secretion of TNF-α and IFN-γ	Activation the immune system Creating a proinflammatory environment	Wei et al., 2020 (19)
	αvβ6-positive tumor cell lines of pancreatic and breast cancer	Murine	Cells expressing high levels of αvβ6 (BxPc, PANC0403, Suit2) were killed more efficiently by oncolytic Ad5_NULL_-A20 than by oncolytic Ad5	Ad5_NULL_-A20-based virotherapies efficiently target αvβ6-integrin-positive tumors	Davies et al., 2021 (20)
	Advanced metastatic tumors	Murine	Increase in CD8+ T cells Reduction of IFN-γ secretion	Specific immunity against tumor	Cerullo et al., 2010 (21)
	Breast cancer	Murine	Inflammation and neutrophil infiltration due to oncolytic adenovirus-GM-CSF.	Ad5/3-D24-GMCSF, combined with low-dose CP showed efficacy and antitumor activity	Bramante et al., 2016 (22)
	Solid tumors	Murine	CD8 cytotoxicity viruses efficiently lysed tumors	Significantly prolonged survival	Gürlevik et al., 2010 (23)
	Metastatic ductal breast cancer	Murine	Each virus featured 5/3 chimerism of a promoter controlling the expression of E1A and fiber, which was also deleted in the Rb binding domain for additional tumor selectivity	These viruses completely eradicated CD44+ low CD24−/cells *in vitro* Significant antitumor activity in CD44+ CD24−/low-derived tumors *in vivo*	Bauerschmitz et al., 2008 (24)
	Metastatic melanoma	*In vitro*	Activation and an increased costimulatory capacity of monocyte-derived antigen-presenting cells	A valuable immunotherapeutic agent for melanoma is ORCA-010	González et al., 2020 (25)
	Gastric cancer MKN45 and MKN7 cells	Murine	Cell death in stem cells such as CD133 resident cancer by stimulating cell-cycle-related proteins	Killing cancer cells	Yano et al., 2013 (26)
Herpesvirus	Bearing M3-9-M tumors	Murine	Increasing the incidence of CD4+ and CD8+ T cells and no correlation with the CD4+CD25+Foxp3+ regulatory T-cell populations in the tumor	An efficient therapy strategy for soft tissue sarcoma in childhood	Chen et al., 2017 (27)
	Breast cancer	Murine	Regulation of CD8+ T cell activation markers in the tumor microenvironment Inhibition of tumor angiogenesis	Tumor regression Anticancer immune response	Ghouse et al., 2020 (28)
	Colon carcinoma	Murine	Decreased inhibitory immune cells Increased positive immune cells in the spleen.	Generate tumor-specific immunity Elimination of primary tumors Developing immune memory to inhibit tumor recurrence and metastasis.	Zhang et al., 2020 (29)
	Ovarian carcinoma	Murine	DC maturation and tumor infiltration of INF-γ+ CTL	The antitumor immune responses are facilitated	Benencia et al. 2008 (30)
	Tumor	Murine	T-cell responses against primary or metastatic tumors	Antitumor immune response Prevention of tumor growth	Li et al., 2007 (31)
	STING low-metastatic melanoma	Murine	Release of DAMP factors Release of IL-1β and inflammatory cytokines Induction of host antitumor immunity	Induction of immunogenic cell death (ICD) Recruitment of viral and tumor-antigen-specific CD8+ T cells STING expression as a predictive biomarker of T-Vec Response	Bommareddy et al., 2019 (32)
	Osteosarcoma cells	Murine	Antitumor efficacy *in vivo* Inducing antitumor immunity	The *in vitro* cytolytic properties of OVs are poor prognostic indicators of effective cancer virotherapy and *in vivo* antitumor activity	Sobol et al., 2011 (33)
	HCT8 human colon cancer cells	Murine	Cytotoxicity, viral replication, and Akt1 expression	Therapy of TIC-induced tumors with NV1066 slowed tumor growth and yielded tumor regression	Warner et al., 2016 (34)
	Glioblastoma-derived cancer stem-like cells (GBM-SC)	Murine	Infection with HSV G47Delta killed GBM-SCs and inhibited their self-renewal and the inability of viable cells to form secondary tumor spheres	Significant anti-tumor effect against xenografts in mice and effective killing of CSCs *in vitro*	Wakimoto et al., 2009 (35)
	Solid tumors	Human	The induction of adaptive antitumor immune responses	All patients were seropositive. No local recurrence was observed in patients and disease-specific survival was 82.4%	Harrington et al., 2010 (36)
	Breast, head and neck, and gastrointestinal cancers, and malignant melanoma	Human	Induction of adaptive anti-tumor immune responses	Biopsies contained residual tumor was observed in 19 patients after treatment that 14 of them showed tumor necrosis (extensive, or apoptosis)	Hu et al., 2006 (37)
	Metastatic melanoma	Human	ICP47 deletion increases US11 expression and enhances virus growth and replication in tumor cells	Overall survival at 12 and 24 months were 58% and 52%, respectively.	Senzer et al., 2009 (38)
Measles virus	Solid tumor	Murine	GOS/MV-Edm significantly increases viral replication in tumor mass	Increased survival in passive antiserum immunized tumor-bearing mice	Xia et al., 2019 (39)
	Orthotopic glioma tumor spheres and primary colon cancer	Murine	Overexpression of the CD133 target receptor or increased kinetics of proliferation through tumor cells	CD133-targeted measles viruses selectively removed CD133þ cells from tumor tissue	Bach et al., 2013 (40)
	Mesothelioma	Murine	Infiltration of CD68+ cells innate immune cells.	Oncolytic MVs is versatile and potent agents for the treatment of human mesothelioma.	Li et al., 2010 (41)
	Multiple myeloma	Murine	Induction of adaptive anti-tumor immune responses	Virus-infected T cells may induce systemic measles virus therapy in the presence of ABS antivirus.	Ong et al., 2007 (42)
	Breast cancer	*In vitro*	Inducing apoptosis	Induction of cell death leads to infection of breast cancer cells with rMV-BNiP	Lal and Rajala et al., 2019 (43)
	Breast cancer	*In vitro*	Increased percentage of apoptotic cells in infected MCF-7 cells	Significant apoptosis in breast cancer cell lines.	Abdullah et al., 2020 (44)
	T-cell lymphomas (CTCLs)	Human	An increase in the IFN-γ/CD4 and IFN-γ/CD8 mRNA ratio and a reduced CD4/CD8 ratio	MV can affect CTCL treatment.	Heinzerling et al., 2005 (45)
Newcastle disease virus	Lung cancer	Murine	Caspase-dependent apoptosis associated with increased caspase-3 processing and ADP-ribose polymerase cleavage.	A potential strategy for targeting lung CSCs	Hu et al., 2015 (46)
	B16 melanoma	Murine	Treatment with systemic CTLA-4 blockade was due to long-term survival and tumor rejection	Distant tumors are prone to systemic therapy with immunomodulatory antibodies using localized therapy with oncolytic NDV	Zamarin et al., 2014 (47)
	Lung cancer	Murine	DAMP release Autophagy induction	Inhibited tumor growth Trigger ICD	Ye et al., 2018 (48)
	GBM	Murine	GBM susceptibility to NDV is dependent on the loss of the type I IFN	Trigger the activation of immune cells against the tumor and show oncolytic effect	García-Romero et al., 2020 (49)
Vaccinia virus	Melanoma	Murine	PD-L1 inhibition Neoantigen presentation	Tumor neoantigen-specific T-cell responses	Wang et al., 2020 (50)
	Solid tumors	Murine	Activated the inflammatory immune status	Complete tumor regression long-term tumor-specific immune memory	Nakao et al., 2020 (51)
	Solid cancer	Murine	Replication was activated by EGFR/Ras pathway signaling, cellular TK levels, and cancer cell resistance to IFNs	Selectively cell lysis and stimulation of antitumoral immunity	Parato et al., 2012 (52)
M1 virus	Melanoma	Murine	CD8^+^ T-cell-dependent therapeutic effects long-term antitumor immune memory Upregulating the expression of PD-L1	Immunogenic tumor cell death Restores the ability of dendritic cells to prime antitumor T cells	Yang Liu et al., 2020 (11)
	Bladder tumor	Murine	Inhibition of CCDC6 improve viral replication and then induced endoplasmic reticulum stress to facilitate M1 virus oncolytic effects.	CCDC6 inhibition resulted in better antitumor activity	Liu et al., 2021 (53)
Poxvirus	MC-38 colon adenocarcinoma tumors	Murine	Elicited TILs with lower quantities of exhausted PD-1^hi^Tim-3^+^ CD8^+^ T cells and regulatory T cells	Tumor regression and improved survival	Mathilde et al., 2020 (54)
Poliovirus	Breast cancer	Murine	Primary oncolytic viral receptors are highly expressed in tumor cells and transmitted among cells.	Oncolytic PV recombinants may affect tumor cells by viral receptor CD155	Ochiai et al., 2004 (55)
Reovirus	Solid tumor	Murine	Induction of Golgi fragmentation and accumulation of oncogenic Ras in the Golgi body	Initiating apoptotic signaling events required for virus release and spread.	Garant et al., 2016 (56)
Adenovirus (Ad), Semliki Forest virus (SFV) and Vaccinia virus (VV)	Osteosarcoma	Murine	Activates immunogenic apoptosis Triggering phagocytosis and maturation of DCs Th1-cytokine release by DCs and antigen-specific T-cell activation.	Induction of T-cell-mediated antitumor immune responses. Increased cell death processes	Jing Ma et al., 2020 (57)

PD-L1, programmed death-ligand 1; Ad, adenovirus; MV, measles virus; GBM, glioblastoma; NDV, Newcastle disease virus; VV, Vaccina virus; Th, T helper; ICD, immunogenic cell death; EGFR, epidermal growth factor receptor; TK, thymidine kinase; IFN-I, type-I interferon; HSV, herpes simplex viruses; TIL, tumor infiltration lymphocyte; DC, dendritic cells; BHV, bovine herpesvirus; DAMP, damage-associated molecular pattern; Trail, TNF-related apoptosis-inducing ligand; GD-55, GOLPH2-regulated oncolytic adenovirus; GOS, graphene oxide arms PV, polio virus; LAPV, Israeli acute paralysis virus; CP, cisplatin; GM-CSF, granulocyte–macrophage colony-stimulating factor.

According to **Table 1**, OVs may employ multifunction against tumor cells; however, the most antitumor actions of OVs were related to cytolysis activity and inducing antitumor immunity (n = 26) in which adenovirus (n = 11) and HSV (n = 9) were the most responsible OVs in their categories, respectively. However, the last action was associated with vascular collapse. The collective data in **Table 2** exhibited a summary of clinical trials of OVs implicated in malignancies highlighting the most considerable focus on engineered VV by TK^del^ GMCSF ^exp^ (JX-594) on solid tumors supported by Jennerex Biotherapeutics Company. The majority of studies under clinical trials involve a transgene virus encoding an immune-stimulatory or proapoptic gene to boost the oncolytic features of the virus. As **Table 2** reveals, granulocyte–macrophage colony-stimulating factor (GM-CSF) and pro-drug-converting enzymes are the most popular transgenes, although many OVs encoding novel therapeutic cargos are in clinical development. Streby et al., in phase I clinical trial, examined the effects of HSV1716 on relapsed/refractory solid tumors. Despite the fact that none of the patients exhibited objective responses, virus replication and inflammatory reactions were seen in patients (58). In another clinical trial, Desjardins et al. reported a higher survival rate in grade IV malignant glioma patients who received recombinant nonpathogenic polio–rhinovirus chimera (59). In a phase I clinical trial, Rocio Garcia-Carbonero et al. discovered that enadenotucirev IV infusion was associated with high local CD8+ cell infiltration in 80% of tumor samples evaluated, indicating a possible enadenotucirev-driven immune response (60). TG4023, a modified vaccinia Ankara viral vector carrying the FCU1 suicide gene, was used in a phase I trial to convert the non-cytotoxic prodrug flucytosine (5-FC) into 5-fluorouracil (5-FU) in the intratumor. Finally, 16 patients with liver tumors were successfully injected; the MTD was not achieved, and a high therapeutic index was demonstrated (61). Dispenzieri et al. examined MV-NIS effects in patients with relapsed, refractory myeloma and reported satisfactory primary results (62).

**Table 2:** The summary of clinical trials for oncolytic viruses.

Phase	Virus	Tumor	Interventions	Trial code	Country	Company
Phase I	JX-594	Refractory solid tumors	Intratumoral injection	NCT01169584	USA	Jennerex Biotherapeutics
	JX-594	Refractory solid tumors	Intravenous infusion	NCT00625456	Canada	Jennerex Biotherapeutics
	HSV-1, TBI-1401 (HF10)	Solid tumor with superficial lesions	Intratumoral administration	NCT02428036	Japan	Takara Bio Inc.
	Recombinant measles virus	Ovarian cancer Primary peritoneal cavity cancer	Intraperitoneal administration	NCT00408590	USA	Mayo Clinic
	GM-CSF-Adenovirus CGTG-102	Malignant solid tumor	In combination with low dose cyclophosphamide	NCT01598129	Finland	Targovax Oy
	Adenovirus VCN-01	Solid tumor	Intravenous administration with or without gemcitabine	NCT02045602	Spain	VCN Biosciences, S.L.
	REOLYSIN^®^	KRAS mutant metastatic colorectal Cancer	Intravenous administration with Irinotecan/Fluorouracil/Leucovorin and Bevacizumab	NCT01274624	USA	Oncolytics Biotech
	Adenovirus VCN-01	Pancreatic cancer	Intratumoral injections with intravenous Gemcitabine and Abraxane^®^	NCT02045589	Spain	VCN Biosciences, S.L.
	JX-594	Hepatic carcinoma	Transdermal injection	NCT00629759	Korea	Jennerex Biotherapeutics
	Attenuated Vaccinia Virus, GL-ONC1	Solid organ cancers	Intravenous administration	NCT00794131	United Kingdom	Genelux Corporation
	Coxsackievirus Type A21	Melanoma	Intratumoural injection	NCT00438009	Australia	Viralytics
	REOLYSIN^®^	Pancreatic adenocarcinoma	Pembrolizumab (KEYTRUDA^®^)	NCT02620423	USA	Oncolytics Biotech
	Vaccinia Virus (GL-ONC1)	Head and neck carcinoma	With concurrent Cisplatin and radiotherapy	NCT01584284	USA	Genelux Corporation
Phase II	TBI-1401(HF10)	Melanoma	In combination with Ipilimumab	NCT03153085	Japan	Takara Bio Inc.
	HF10	Malignant melanoma	With Ipilimumab	NCT02272855	USA	Takara Bio Inc.
	OncoVEX^GM-CSF	Melanoma	Intratumoral injection	NCT00289016	United Kingdom	–
	Edmonston strain of Measles Virus Expressing NIS	Refractory multiple myeloma	Systemic Administration with cyclophosphamide	NCT02192775	USA	University of Arkansas
	Reovirus Serotype 3 REOLYSIN^®^	Non-small cell lung cancer	Intravenous administration with paclitaxel and carboplatin	NCT00861627	USA	Oncolytics Biotech
	JX-594	Hepatocellular carcinoma	Intratumoral injection	NCT00554372	USA	Jennerex Biotherapeutics
	CG0070	Non-muscle invasive bladder carcinoma	–	NCT02365818	USA	CG Oncology, Inc.
	Wild-type Reovirus REOLYSIN^®^	Bone and soft tissue sarcomas	Intravenous injection	NCT00503295	USA	Oncolytics Biotech
Phase I/II	Vaccinia Virus JX-594	Melanoma	Intratumoral injection	NCT00429312	USA	Jennerex Biotherapeutics
	Parvovirus H-1	Glioblastoma multiforme	Intratumoral/Intracerebral injection	NCT01301430	Germany	Oryx GmbH & Co. KG
	HSV1716	Malignant pleural mesothelioma	Intrapleural injection	NCT01721018	United Kingdom	Virttu Biologics Limited
	Ad-MAGEA3	Metastatic non-small cell lung cancer	With pembrolizumab	NCT02879760	Canada	Turnstone Biologics, Corp.
	REOLYSIN^®^	Recurrent malignant gliomas	Intralesional administration	NCT00528684	USA	Oncolytics Biotech
	JX 594	Colorectal carcinoma	Multiple intravenous with Irinotecan	NCT01394939	USA	Jennerex Biotherapeutics
	Vaccinia Virus GL-ONC1	Peritoneal Carcinomatosis	Intraperitoneal administration	NCT01443260	Germany	Genelux GmbH

Cohn et al., in phase II clinical trial, evaluated the effects of oncolytic reovirus (Reolysin^®^) plus weekly paclitaxel in women with recurrent or persistent ovarian, tubal, or primary peritoneal cancer. The results did not show any improvement in the patient status (63), although Mahalingam et al. showed that REOLYSIN^®^, plus carboplatin and paclitaxel, is an effective treatment in advanced malignant melanoma (64). Packiam et al. showed that CG0070 (GM-CSF expressing adenovirus) has a 47% CR rate at 6 months for all patients and 50% for patients with carcinoma-*in situ* (65).

Geletneky et al. evaluated H-1 parvovirus (H-1PV) effects in recurrent glioblastoma patients and reported microglia/macrophage activation and cytotoxic T-cell infiltration in the infected tumors, proposing initiation of the immunogenic response (66).

Andtbacka et al., in a phase III study, evaluated Talimogene laherparepvec (T-VEC) in stage IIIc and stage IV malignant melanoma. T-VEC was the first approved OVs against melanoma in a phase III clinical trial. This virus compared with GM-CSF showed a higher durable response rate and overall survival (67). In another newest phase III study, Talimogene laherparepvec was approved by the Food and Drug Administration (FDA) in the USA, European Union, and Australia (68).

## Discussion

**Figure 1:**
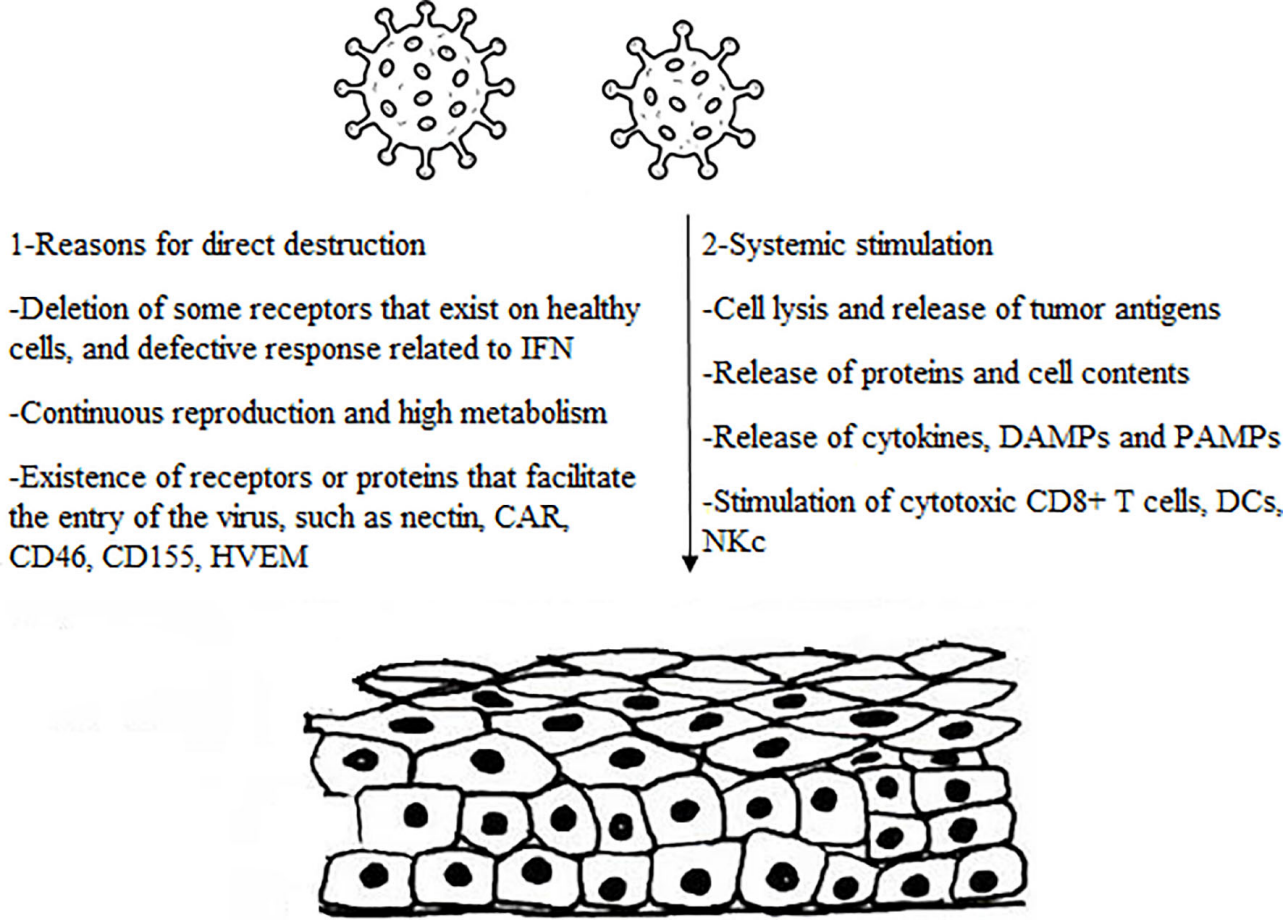
The main mechanism involved by oncolytic viruses.

As a challenge in cancer therapy approaches (1), the exclusive features of oncolytic viruses have attracted plenty of researchers in recent years. OVs have the dramatic capability to selectively infect tumor cells leading to direct or indirect cancer cell death without harming normal cells (7). This study focused on some mechanisms employed by OVs against tumor cells, which are exactly various from virus to virus (**Figure 1**).

According to most studies, OVs can target cancer cells and benefit from tumor conditions in favor of replication in infected cells, eventually leading to oncolysis. Indeed, tumor cells tend to resist apoptosis and translational suppression, which are both compatible with the growth of several viruses (7). One of the main actions of OVs is to take advantage of immune-evading properties of cancer cells to escape from recognition and destruction by the immune system. Antiviral processes in normal cells are associated with the interferon pathway in which the secretion of type I interferon (IFN) cytokine can trigger an antiviral response and induce ISGs to block viral replication (69). This subsequently leads to cell apoptosis, as it is known that the IFN-I signaling regulates the expression of proapoptotic genes such as tumor necrosis factor alpha (TNF-α), FAS ligand, and tumor necrosis factor-related apoptosis-inducing ligand (TRAIL) (70).

Regarding the IFN-I signaling is defective in most tumor cells, it makes tumor cells susceptible to being infected by some OVs including NDV, VSV, MYXV, and raccoon pox virus (71–73). García-Romero et al. showed that NDV was able to replicate in glioblastoma (GBM) cancer stem cells (CSCs) due to type I IFN gene loss occurring in more than 50% of patients. Infection of GBM with NDV represents oncolytic and immunostimulatory properties through the production of type I IFN in non-tumor cells such as tumor infiltrated macrophages and DC or other cells present at the tumor microenvironment (49). NDV therapy also declines CSCs self-renewing capacity to improve their differentiation ability and facilitate cancer therapy (49, 74). OVs can also benefit from the abnormal expression of the proto-oncogene RAS which generally occurs in normal cells but actives in tumor cells (75). OV infection outcomes can be affected by up-regulation of RAS in tumoral cells and further down-regulation of interferon-inducible genes due to activation of RAS/MEK signaling pathway that reduces viral response in tumoral cells (76). On the contrary with this attempt, Garant et al. demonstrated that reovirus could translocate and accumulate RAS into Golgi apparatus to increase apoptotic signaling events required for virus release (56). This highlighted that the outcomes of OVT are exclusively associated with the characteristics and type of OVs.

High expression of some viral receptors by cancer cells permits higher viral uptake in cancer cells than in normal ones. Some receptors such as CAR (77), laminin (78), CD155 (79), and CD46 (80) are overexpressed in various cancer cells which result in increased uptake of Ad (81), Sindbis virus (82), PV (83), and MV (84) respectively. Interestingly, some viral proteins are poisonous for neoplastic cells and can directly kill cells before viral replication. This was evidenced by the E3 death protein and E4orf4 proteins encoded by Ads and are toxic for cells that end in cytolysis at the time of virus exposure (3). However, deletion in specific viral genes can be another mechanism for the action of the OVs. These genes are necessary for the longevity of viruses in normal cells but not essential for viral activity in cancer cells. Thymidine kinase (TK) is an indispensable enzyme for nucleic acid metabolism encoded in infection with wild type vaccinia virus and enables the replicating of the virus in normal cells. Lister strain virus with TK gene deletion as a type of VV has shown a beneficial antitumor potency and cancer-selective replication *in vivo* since tumoral cells have a high TK content, which enables the virus to replicate in cancer cells regardless of the deletion in viral TK gene (85). In parallel with this study, Parato et al. analyzed the mechanism of cancer-selectivity by an engineered vaccina virus with TK deletion and epidermal growth factor (EGFR) and lac-Z transgenes observing the replication in tumor cells was related to activation of EGFR/RAS signaling, high cellular TK level and tumor cell resistance to IFN-I (52). These results displayed noticeably the beneficial implication of OVs with inherent and engineered mechanistic properties in cancer therapy approaches.

Oncolytic viruses may interfere with normal physiological process of tumor cells to induce the secretion of pro-inflammatory mediators or even lead to the exposure of tumor-associated antigens (TAA), pathogen-associated molecular patterns (PAMPs) and damage-associated molecular patterns (DAMPs) following apoptosis or oncolysis. These responses can also result in a change in tumor status from immune desert to inflamed status and further recruit a collection of immune cells such as cytotoxic T lymphocytes, dendritic cells, natural killer cells and phagocytic cells to induce immune cell death along with antiviral responses (86, 87).

Remarkably, most viruses continue their infection by expressing genes responsible for escaping the immune system and disseminating in host cells (88). Mutation in these genes can probably improve immune induction and thus increase the anti-tumoral responses regardless these mutations may reduce virus replication further (10). Thus, oncolytic viruses are often engineered to express various genes aided in the overall anti-tumor efficacy of the virus. Transgenes mostly include ranging from immune-stimulatory (IL-2, IL-4, IL-12 and GM-CSF) to pro-apoptotic (tumor necrosis factor alpha, p53 and TRAIL genes inserted into oncolytic viruses (87, 89–94). Interestingly, bystander effects of OVs through local release of cytokines can potentially cause immune response against nearby tumor cells even without direct antigen expression (95).

Furthermore, OVs can destroy tumor vasculature and impede sufficient intratumoral blood reserve, which is essential for tumor progression and metastasis (96). Breitbach et al. demonstrated that intravenous injection of JX-594, an engineered vaccine virus with TK deletion and overexpression of human granulocyte-monocyte colony-stimulating factor (hGM-CSF), led to replication of the virus in endothelial cells of the nearby tumor and disrupted tumor blood flow, which ultimately ended in intensive tumor necrosis within 5 days. Consistently, patients with advanced hepatocellular carcinoma, hypervascular and VEGF^high^ tumor type, treated by JX-594 in phase II clinical trials confirmed the efficiency of the JX-594 OV in tumor vasculature disruption without toxicity to normal blood vessels in which inhibition of angiogenesis can passively result in tumor regression (97). This evidence may open promising technologies toward cancer therapy in a way tumor cells are targeted selectively and bypass the side effects of conventional approaches.

Recently, conditionally replication-competent adenoviruses (CRCAs) have been introduced as a successful method for cancer therapy. Sarkar et al. showed that Ad.PEG-E1A-mda-7, a cancer terminator virus (CTV), selectively replicated in cancer cells, inhibits their growth and induces apoptosis (98).

Qian et al. showed that ZD55 expressing melanoma differentiation-associated gene-7/interleukin-24 (ZD55-IL-24) affects B-lymphoblastic leukemia/lymphoma through upregulation of RNA-dependent protein kinase R, enhance phosphorylation of p38 mitogen-activated protein kinase, and induce of endoplasmic reticulum (ER) stress (99).

Azab et al. showed that Ad.5/3-CTV potently suppressed *in vivo* tumor growth in mouse (100).

Bhoopathi showed that Ad.5/3-CTV induces apoptosis through apoptosis-inducing factor (AIF) translocation into the nucleus, independent of the caspase-3/caspase-9 pathway (101).

In an interesting study, Bhoopathi et al. introduced a novel tripartite CTV “theranostic” adenovirus (TCTV) that targets virus replication, cytokine production, and imaging capabilities uniquely in cancer cells. This TCTV permits targeted treatment of tumors while monitoring tumor regression, with the potential to simultaneously detect metastasis due to the cancer-selective activity of reporter gene expression (102).

Greco et al. showed that ultrasound (US) contrast agents guided MB/Ad.*mda*-7 complexes to DU-145 cells successfully and eradicated not only targeted DU-145/Bcl-xL-therapy-resistant tumors but also nontargeted distant tumors (103).

T-VEC, adenovirus, and vaccinia virus are the most popular OVs in clinical trials. Approving T-VEC by FDA for the first time could pave the way for other OVs in the clinic. Oncolytic viruses have a broad therapeutic method; hence, their clinical development requires a multidisciplinary view. It is necessary to understand viral generation and viability in infected cells. To improve clinical trials, important factors such as viral entrance, replication, dissemination, oncolysis, and immune activation should be controlled. These factors can vary between tumor types and OVs. It is also critical to understand the immune composition of diverse cancers and the immunological repercussions of viro-immunotherapy.

## Conclusion and Future Direction

Cancer is among the most important causes of mortality worldwide, and many chemotherapies and radiotherapy approaches do not have a specific effect on cancer cells and are sometimes accompanied by side effects. Today, a biological war has evolved against cancer by genetically modifying natural pathogens to activate them against neoplastic cells. OVT is a promising therapeutic option in cancer therapy. The mechanisms of action of OVs differ entirely from the mechanism of action of chemotherapy, radiotherapy, surgery, and embolization. They can result in success in the treatment of cancers that are resistant to other therapeutic modalities. Better understanding and acquiring comprehensive information regarding OV therapy and the biology of cancer is an essential step in assessing and controlling cancer programs. 

## Author Contributions

Conceptualization, WK and HE. Methodology, MF and RD. Validation, BJ. Data curation, MB. Writing—original draft preparation, HE and WK. Writing—review and editing, all. All authors have read and agreed to the published version of the manuscript.

## Funding

This study was fully sponsored by Applied Virology Research Center; Baqiyatallah University of Medical Science; Tehran; Iran.

## Conflict of Interest

The authors declare that the research was conducted in the absence of any commercial or financial relationships that could be construed as a potential conflict of interest.

## Publisher’s Note

All claims expressed in this article are solely those of the authors and do not necessarily represent those of their affiliated organizations, or those of the publisher, the editors and the reviewers. Any product that may be evaluated in this article, or claim that may be made by its manufacturer, is not guaranteed or endorsed by the publisher.
